# Impact of Glutathione Peroxidase-1 Deficiency on Macrophage Foam Cell Formation and Proliferation: Implications for Atherogenesis

**DOI:** 10.1371/journal.pone.0072063

**Published:** 2013-08-22

**Authors:** Fei Cheng, Michael Torzewski, Adriana Degreif, Heidi Rossmann, Antje Canisius, Karl J. Lackner

**Affiliations:** 1 Institute of Clinical Chemistry and Laboratory Medicine, University Medical Center, Johannes Gutenberg-University, Mainz, Germany; 2 Department of Laboratory Medicine, Robert Bosch-Hospital, Stuttgart, Germany; King’s College London School of Medicine, United Kingdom

## Abstract

Clinical and experimental evidence suggests a protective role for the antioxidant enzyme glutathione peroxidase-1 (GPx-1) in the atherogenic process. GPx-1 deficiency accelerates atherosclerosis and increases lesion cellularity in ApoE^−/−^ mice. However, the distribution of GPx-1 within the atherosclerotic lesion as well as the mechanisms leading to increased macrophage numbers in lesions is still unknown. Accordingly, the aims of the present study were (1) to analyze which cells express GPx-1 within atherosclerotic lesions and (2) to determine whether a lack of GPx-1 affects macrophage foam cell formation and cellular proliferation. Both *in situ*-hybridization and immunohistochemistry of lesions of the aortic sinus of ApoE^−/−^ mice after 12 weeks on a Western type diet revealed that both macrophages and – even though to a less extent – smooth muscle cells contribute to GPx-1 expression within atherosclerotic lesions. In isolated mouse peritoneal macrophages differentiated for 3 days with macrophage-colony-stimulating factor (MCSF), GPx-1 deficiency increased oxidized low density-lipoprotein (oxLDL) induced foam cell formation and led to increased proliferative activity of peritoneal macrophages. The MCSF- and oxLDL-induced proliferation of peritoneal macrophages from GPx-1^−/−^ApoE^−/−^ mice was mediated by the p44/42 MAPK (p44/42 mitogen-activated protein kinase), namely ERK1/2 (extracellular-signal regulated kinase 1/2), signaling pathway as demonstrated by ERK1/2 signaling pathways inhibitors, Western blots on cell lysates with primary antibodies against total and phosphorylated ERK1/2, MEK1/2 (mitogen-activated protein kinase kinase 1/2), p90RSK (p90 ribosomal s6 kinase), p38 MAPK and SAPK/JNK (stress-activated protein kinase/c-Jun N-terminal kinase), and immunohistochemistry of mice atherosclerotic lesions with antibodies against phosphorylated ERK1/2, MEK1/2 and p90RSK. Representative effects of GPx-1 deficiency on both macrophage proliferation and MAPK phosphorylation could be abolished by the GPx mimic ebselen. The present study demonstrates that GPx-1 deficiency has a significant impact on macrophage foam cell formation and proliferation via the p44/42 MAPK (ERK1/2) pathway encouraging further studies on new therapeutic strategies against atherosclerosis.

## Introduction

Glutathione and the glutathione peroxidases (GPxs) constitute the principal antioxidant defense system in mammalian cells [1]. There are four known GPxs containing selenocysteine at the active site. GPx-1 is the ubiquitous intracellular form and major antioxidant enzyme within many cells [2], [3]. While it definitely protects against reactive-oxygen-species (ROS)-induced oxidative stress *in vivo* [4], its role in coping with reactive nitrogen species (RNS) is more complex and probably dependent on the respective cell type [5], [6], [7]. In the absence of this enzyme, resulting reductions in antioxidant defense, which lead to increased ROS accumulation, can elicit numerous pathophysiological consequences [8].

Oxidative stress plays an important role in atherogenesis, amongst others by stimulating oxidized low-density lipoprotein (oxLDL) - induced macrophage cholesterol accumulation and foam cell formation, the hallmark of atherosclerotic lesions [9]. GPxs and reduced glutathione play an important protective role against cell-mediated oxidation of LDL. For example, a recent *in vitro* study showed that reduced GPx-1 expression increased cell-mediated oxidation of LDL [10]. More importantly, clinical evidence also suggests a protective role for GPx-1 in the atherogenic process. Accordingly, a low activity of red blood cell GPx-1 is associated with an increased risk of cardiovascular events in patients with coronary artery disease [11], and carotid atherosclerotic plaques of patients have reduced GPx-1 activity [12]. Recently, an increased expression of several antioxidant enzymes, in particular GPx-1, in the aorta of apolipoprotein E-deficent (ApoE^−/−^) mice during prelesional stages was reported [13]. A mouse model of GPx-1 deficiency provided a new tool for future studies to clarify the mechanisms of its protective function in atherogenesis. Thus, GPx-1 knock-out mice have been shown to have an endothelial dysfunction [14], an effect that is even aggravated by hyperhomocysteinemia [15]. GPx-1 deficiency causes structural alterations in the arterial vessel wall, such as neointima formation and periadventitial inflammation [14]. Finally, our own previous work [16] as well as work by others [17] showed that deficiency of GPx-1 accelerates and modifies atherosclerotic lesion progression in non-diabetic and diabetic ApoE^−/−^ mice.

We have previously also shown that GPx-1 deficiency led to modified atherosclerotic lesions with increased cellularity and that peritoneal macrophages from double-knockout mice showed increased *in vitro* proliferation in response to macrophage colony stimulating factor (MCSF) [16]. However, the origin of GPx-1 within the atherosclerotic lesion as well as its impact on signal transduction pathways responsible for increased cellular proliferation of macrophages is still unknown. Accordingly, the aims of the present study were (1) to identify the cellular distribution of GPx-1 within atherosclerotic lesions and (2) to determine whether a lack of GPx-1 impacts on macrophage foam cell formation and known signal transduction pathways implicated in cellular proliferation.

## Materials and Methods

### Mice

GPx-1^−/−^ mice (generously provided by Ye-Shi Ho, Department of Biochemistry, Wayne State University, Detroit, Michigan, USA) were bred by generating F2 hybrids from the ApoE^−/−^ and GPx-1^−/−^ parental strains. The GPx-1^−/−^ApoE^−/−^ strain could then be propagated successfully by incrossing. Genotype determination was performed as described [14].

### Materials

Recombinant murine MCSF was purchased from PeproTech (Biozol GmbH, Eching, Germany). PD98059, U0126 and ebselen were obtained from Calbiochem (EMD Chemicals, Inc. Merck KGaA, Darmstadt, Germany). Monoclonal rabbit anti-GPX1 (clone EPR3312) antibody for immunohistochemistry was purchased from Novus Europe (Cambridge, UK), monoclonal mouse anti-smooth muscle α-actin (Clone 1A4) antibody for immunohistochemistry was purchased from Dako Cytomation (DakoCytomation Denmark A/S, Glostrup, Denmark). Polyclonal goat anti-apolipoprotein B antibody, monoclonal rat anti-F4/80 (clone CI:A3-1) antibody, polyclonal rabbit antibody to PCNA (proliferating cell nuclear antigen), polyclonal rabbit antibody to phospho-MEK1/2 (MAP2K1/2 pSer217/221), polyclonal rabbit antibody to phospho-ERK1/2 (p44/42 MAPK pThr202) and polyclonal rabbit antibody to phospho-p90RSK1 (RPS6KA1 pThr348) for immunohistochemistry were purchased from Acris Antibodies GmbH (Herford, Germany). A biotin-conjugated monoclonal anti-rabbit IgG antibody was obtained from Sigma (Sigma-Aldrich, St. Louis, USA) and an anti-rat IgG antibody was obtained from Vector Laboratories (Burlingham, CA). Rabbit anti-phospho-ERK1/2, anti-ERK1/2 (extracellular-signal regulated kinase 1/2), anti-phospho-MEK1/2, anti-MEK1/2 (mitogen-activated protein kinase kinase 1/2), anti-phospho-p90RSK, anti-RSK1/2/3 (p90 ribosomal s6 kinase), anti-phospho-p38 MAPK, anti-p38 MAPK (p38 mitogen-activated protein kinase), anti-phospho-SAPK/JNK, anti-SAPK/JNK (stress-activated protein kinase/c-Jun N-terminal kinase) and anti-ß-actin antibodies for Western blots were purchased from New England Biolabs GmbH, Frankfurt, Germany. An alternative anti-actin antibody (for Western blots using the anti-phospho-MEK1/2, anti-MEK1/2, anti-phospho-SAPK/JNK and anti-SAPK/JNK antibodies) and a peroxidase-conjugated anti-rabbit IgG were obtained from Sigma (Sigma-Aldrich, Inc. St. Louis, MO, USA).

### Induction of Atherosclerosis

Female ApoE^−/−^ as well as GPx-1^−/−^ApoE^−/−^ mice were placed on different diets: on a standard chow diet for 5 months for *in vitro* experiments, or on an atherogenic Western-type diet (WTD) at 8 weeks of age for another 12 weeks for *in vivo* experiments. Mice were kept in accordance with standard animal care requirements, housed 4 to 5 per cage, and maintained on a 12 hours light-dark cycle. Water and food were given *ad libitum*.

All animal work performed in this study was conducted according to the national guidelines and was reviewed and confirmed by an institutional review board/ethics committee headed by the local animal welfare officer (Prof. Kempski) of the University Medical Center (Mainz, Germany). The animal experiments were finally approved by the responsible national authority, which is the National Investigation Office Rheinland-Pfalz (Koblenz, Germany). The Approval ID assigned by this authority is AZ 23 177-07/G 07-1-003.

### Tissue Preparation

At the end of the WTD diet, the mice were sacrificed and perfused through the aorta with 4% paraformaldehyde-PBS (PFA). For both immunohistochemistry and *in situ*-hybridization hearts and aortae were resected en bloc down to the iliac bifurcation, fixed in 4% PFA for 12 hours and the aortic arch as well as the aortic sinus were cut in sections as described [18], [19].

### Synthesis of Radiolabeled RNA Probes

Gene specific primers were chosen from published coding sequence of mice GPx-1 (forward: 5′ - AGT ATG TGT GCT GCT CGG CTC T - 3′, reverse: 5′ - CCA GTA ATC ACC AAG CCA ATG C - 3′). cDNA was amplified and the resulting PCR products were cloned in GPx-1-pCR2.1TOPO vector, transformed and amplified in XL10-Gold using TOPO TA Cloning Kit (Invitrogen GmbH, Karlsruhe, Germany). Plasmid DNA was isolated by Plasmid Mini Kit (Qiagen GmbH, Hilden, Germany) and linearized with restriction endonuclease BamHI (New England Biolabs Inc., Ipswich, USA). Sense and anti-sense cRNA were transcribed from linearized plasmid templates using T7 RNA polymerase MAXIscript in vitro Transcription Kit (Ambion Inc., Austin, USA) and [α-^33^P] UTP (GE Healthcare Europe GmbH, Freiburg, Germany). The sense probe was used in parallel as a negative control.

### *In situ*-Hybridization

The ^33^P labeled sense and antisense GPx-1 cRNA were hybridized to the tissue sections. Treatments of the sectioned material were carried out as described by mRNAlocator In Situ Hybridization Kit protocol (Ambion Inc., Austin, USA). Hybridization temperature for GPx-1 was 55°C. After posthybridization treatment, the slides were dipped in autoradiographic Hypercoat LM-1 emulsion (GE Healthcare, Buckinghamshire, UK) and exposed for 3 weeks at 4°C in the dark. After development and fixation, the slides were counterstained with hemalaun and mounted.

### Histochemistry and Immunohistochemistry

Serial 5 µm-thick sections of the paraffin-embedded aortic arch and aortic sinus were deparaffinized in xylene and alternately stained with trichrome or used for immunohistochemistry. Immunostaining of murine tissues with the murine MAbs was performed using the M.O.M. Elite Peroxidase Kit, and the staining with rat or rabbit antibodies was performed using the VECTASTAIN Elite ABC Kit (Vector Laboratories, Burlingham, CA). The following antibodies and dilutions were used: rabbit anti-GPX1 (clone EPR3312, 1∶100), murine anti-smooth muscle α-actin (Clone 1A4, 1∶100), rat anti-F4/80 (Clone CI:A3-1, 1∶100), goat anti-apolipoprotein B (10 µg/ml), rabbit anti-PCNA (1∶100), rabbit anti-phospho-MEK1/2 (MAPK2K1/2 pSer217/221, 1∶50), rabbit anti-phospho-ERK1/2 (p44/42 MAPK pThr202, 1∶50) and rabbit anti-phospho-p90RSK1 (RPS6KA1 pThr348, 1∶50). The reaction products were revealed by immersing the slides in diaminobenzidine tetrachloride (DAB) to give a brown reaction product. For double staining, slides were incubated with the first antibody and the reaction was developed with the Vectastain ABC kit and DAB (Vector Laboratories, Burlingame, CA). The reaction with the second primary antibody was developed with the Vectastain ABC-AP kit (Vector Laboratories, Burlingame, CA) and Liquid Permanent Red Substrate-Chromogen (Dako Deutschland GmbH, Hamburg, Germany) to give a red-colored reaction product. The slides were then counterstained with hemalaun and mounted. Negative controls included replacement of the primary antibody by irrelevant isotype-matched antibodies.

A categorical scoring system was adopted for visual interpretation of the serial slices to allow semiquantitative analysis of immunohistochemistry for PCNA and phospho-ERK1/2 in both GPx-1^−/−^ApoE^−/−^ mice and ApoE^−/−^ mice. The proportion of the positively stained area stained relative to the total lesion area (designated as 100%) was assigned to 1 of 5 scores: 0, 0 to 5%; 1, 6% to 25%; 2, 26% to 50%; 3, 51% to 75%; or 4, 76% to 100%. Statistical analysis was performed by χ^2^ test for categorical variables [20].

### Isolation of Peritoneal Macrophages

Peritoneal macrophages from GPx-1^−/−^ApoE^−/−^ and ApoE^−/−^ mice were prepared by intraperitoneal injection of 1 ml 3% thioglycollate (Fluka, Sigma-Aldrich, Inc. St. Louis, MO, USA). After 4 days, cells were harvested by intraperitoneal lavage with 8 ml DMEM (Dulbecco’s Modified Eagle Medium, Biochrom, Berlin, Germany) and centrifuged for 5 minutes at 460×g. The pellet was resuspended in DMEM supplemented with 10% fetal calf serum (PAA Laboratories, Pasching, Austria), L-glutamine, penicillin/streptomycin (GIBCO, Invitrogen Ltd, Paisley, UK) and plated in cell culture dishes. After incubation for 3 hours, nonadherent cells were removed. The thioglycollate-elicited peritoneal macrophages were incubated for 3 days with MCSF (10 ng/ml).

### Isolation and Modification of LDL

Low-density lipoprotein (LDL) from healthy subjects, aged 18 to 65 years, was isolated by preparative ultracentrifugation. Cholesterol was determined using Chol assay (Abott Laboratories, IL, USA). Concentrations given refer to total cholesterol concentration in the lipoprotein samples. oxLDL was prepared as described [21].

### Lipid Staining with Oil-Red O

After 24 hours of incubation with oxLDL and MCSF, the cells were fixed with 4% PFA, stained with a saturated solution of oil-red O (Sigma, Sigma-Aldrich, Inc. St. Louis, MO, USA) in 60% isopropanol an then counterstained with hemalaun as previously described [22].

### Measurement of Cellular Cholesterol

Cellular content of total cholesterol was quantified by a Cholesterol/Cholesteryl Ester Quantitation Kit (Calbiochem, Merck KGaA, Darmstadt, Germany). After 24 hours of treatment with oxLDL, cells were lysed and homogenized in an ice-cold cholesterol reaction buffer from the kit. Cell lysates were used as sample proteins. Fluorescence was measured in a Fluoroskan microplate reader (Thermo Labsystems, Waltham, USA) with an excitation wavelength of 530 and an emission wavelength of 620 nm. Protein concentrations were determined by the DC protein assay (Biorad, Hercules, USA). The results were expressed as total cholesterol per cellular protein (µg cholesterol/mg protein).

### RNA-isolation and Quantitative Real-Time PCR

After 24 hours of incubation with oxLDL and MCSF, total RNA from macrophages was isolated using the RNeasy Mini Kit (Qiagen, Hilden, Germany). DNA digestion was performed using the RNase-Free DNase Set from Qiagen. Reverse transcription of total RNA was done with Oligo-dT-Primer and the SuperScript III Reverse Transcriptase Kit (Invitrogen, Karlsruhe, Germany). Expression of scavenger receptors CD36 and SRA was measured by quantitative real-time PCR (qRT-PCR) and normalized to ribosomal ribonucleic acid 18S (18S rRNA). qRT-PCR was performed using the LightCycler Real-Time PCR Detection system (Roche Diagnostic, Penzberg, Germany) with QuantiTect SYBR Green PCR Kit (Qiagen). The following primers were used for PCR amplification: CD36 forward 5′ - TTC GCT TCC ACA TTT CCT ACA T - 3′, reverse 5′ - ATG GTC CCA GTC TCA TTT AGC C - 3′; SRA1 forward 5′ - GGG AAC ACT CAC AGA CAC TGA AA - 3′, reverse 5′ - GGG TTG ATC CGC CTA CAC TC - 3′ and 18S rRNA forward 5′ - GAT ACC GCA GCT AGG AAT AAT G - 3′, reverse: 5′ - GCG CAA TAC GAA TGC C - 3′.

### Proliferation Assay

After incubation with MCSF for 3 days, cells were detached with 2 ml Accutase (PAA Laboratories, Pasching, Austria) and plated in a 96-well plate (2.5×10^4^ cells/well). Cells were incubated with MCSF or oxLDL and BrdU for another 48 h. Furthermore, cells with pretreament of ERK-inhibitors (PD98059, U0126) or the GPx mimic ebselen were incubated with MCSF or oxLDL and BrdU for another 48 hours. The proliferation assay (Roche Diagnostics, Mannheim, Germany) was performed according to the manufacturer’s instruction. Briefly, after fixation and permeabilization of the cells, the incorporated BrdU was detected by an anti-BrdU-POD antibody followed by incubation with Luminol. The amount of bound antibody was quantified by determination of relative light units with a chemiluminescence plate reader (Fluoroskan; Thermo Labsystems, Waltham, USA). The results were expressed as the relative proliferation rate relating to control cells from ApoE^−/−^ mice without stimulus.

### Western Blot

After treatment with MCSF with or without ebselen or oxLDL, cells were lysed in an ice-cold buffer containing 1% SDS (Sodium Dodecyl Sulfate), 50 mM Tris-HCl, 5 mM EDTA and 1∶10 diluted Protease Inhibitor Cocktail (Sigma, Steinheim, Germany). Cell lysates were used as sample proteins. Protein concentrations were determined by the DC protein assay (Biorad, Hercules, USA). Samples (0.4 mg/ml) were applied to 10% SDS gels, separated by electrophoresis, and then transferred to nitrocellulose membranes (Hybond-ECL, Amersham Biosciences Ltd, Bucks, UK) according to standard procedures. After blocking of nonspecific sites, membranes were incubated with primary antibodies against total and phosphorylated MEK1/2, ERK1/2, p90RSK, p38 MAPK and SAPK/JNK as well as ß-Actin or Actin (1∶1000). After washing, the membranes were stained with horseradish peroxidase conjugated goat anti-rabbit IgG secondary antibodies, and detected by enhanced chemiluminescence reagent (Amersham, GE Healthcare Ltd, Buckinghamshire, UK). The relative intensity of bands was analyzed by scanning the film and subsequent quantification by Quantity One Software (Biorad, Hercules, USA). Quantitative results of phosphorylated MEK1/2, ERK1/2, p90RSK, p38 MAPK and SAPK/JNK were normalized for the levels of total MEK1/2, ERK1/2, RSK1/2/3, p38 MAPK and SAPK/JNK. ß-Actin (45 kD) or Actin (42 kD) were used as controls.

### Statistical Analysis

All data were expressed as mean ± SD of at least three independent experiments. Every experiment was measured in triplicate for each concentration, time point and/or stimulus/inhibitor (except for Western blot analysis). The outcome parameters determined in this study were analyzed by the Student t test. The results of the proliferation assays did not follow a normal distribution as judged by the Shapiro-Wilk test, thus statistical analyses were performed with the non-parametric Mann-Whitney U test. Differences were considered statistically significant at p<0.05.

## Results

### Localization of GPx-1 and Lipoproteins in Mice Atherosclerotic Lesions

**Figure 1:**
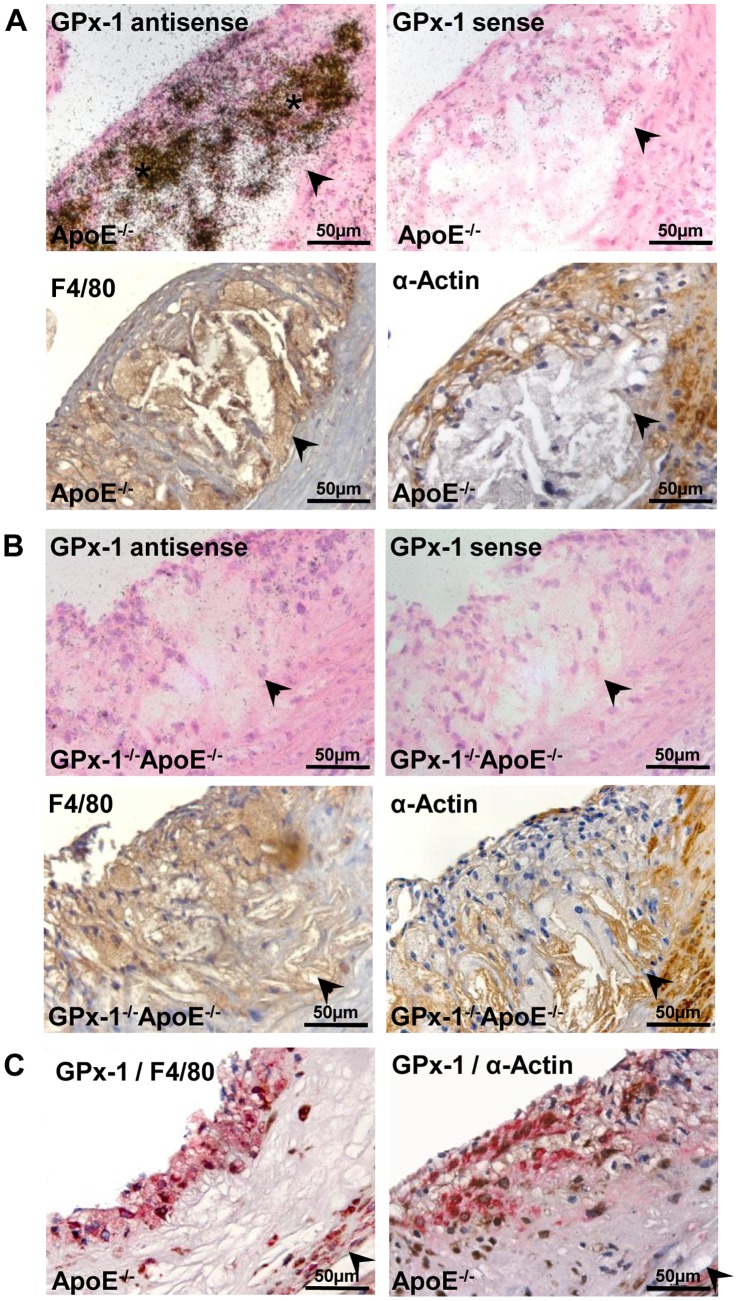
Localization of GPx-1 in mice atherosclerotic lesions. GPx-1 mRNA and protein expression, macrophages and SMCs in sequential sections of the aortic sinus of ApoE^−/−^ (**A, C**) and GPx-1^−/−^ApoE^−/−^ (**B**) mice. **A,** GPx-1 mRNA expression was detected by *in situ*-hybridization (upper panels) and both macrophages and SMCs were detected by immunohistochemistry (middle panels, see Methods). Upper left panel: anti-sense probe; upper right panel: corresponding section hybridized with the sense probe for GPx-1 (no signal). **B,** Control sections of GPx-1^−/−^ApoE^−/−^ mice showed no expression of GPx-1 mRNA, neither with the anti-sense nor with the sense probe (upper panels). The representative atherosclerotic lesion containes both macrophages (lower left panel) and SMCs (lower right panel). **C,** Representative double immunohistochemical staining for GPx-1 (brown) and macrophages (red; left panel) and GPx-1 (brown) and SMCs (red; right panel) in ApoE^−/−^ mice. Note the close intermingling and overlapping of the different antigens predominantly within the inner parts of the intima. In **A** to **C**, the vessel lumen is to the upper left-hand corner. The demarcation between intima and media is indicated by arrowheads.

**Figure 2:**
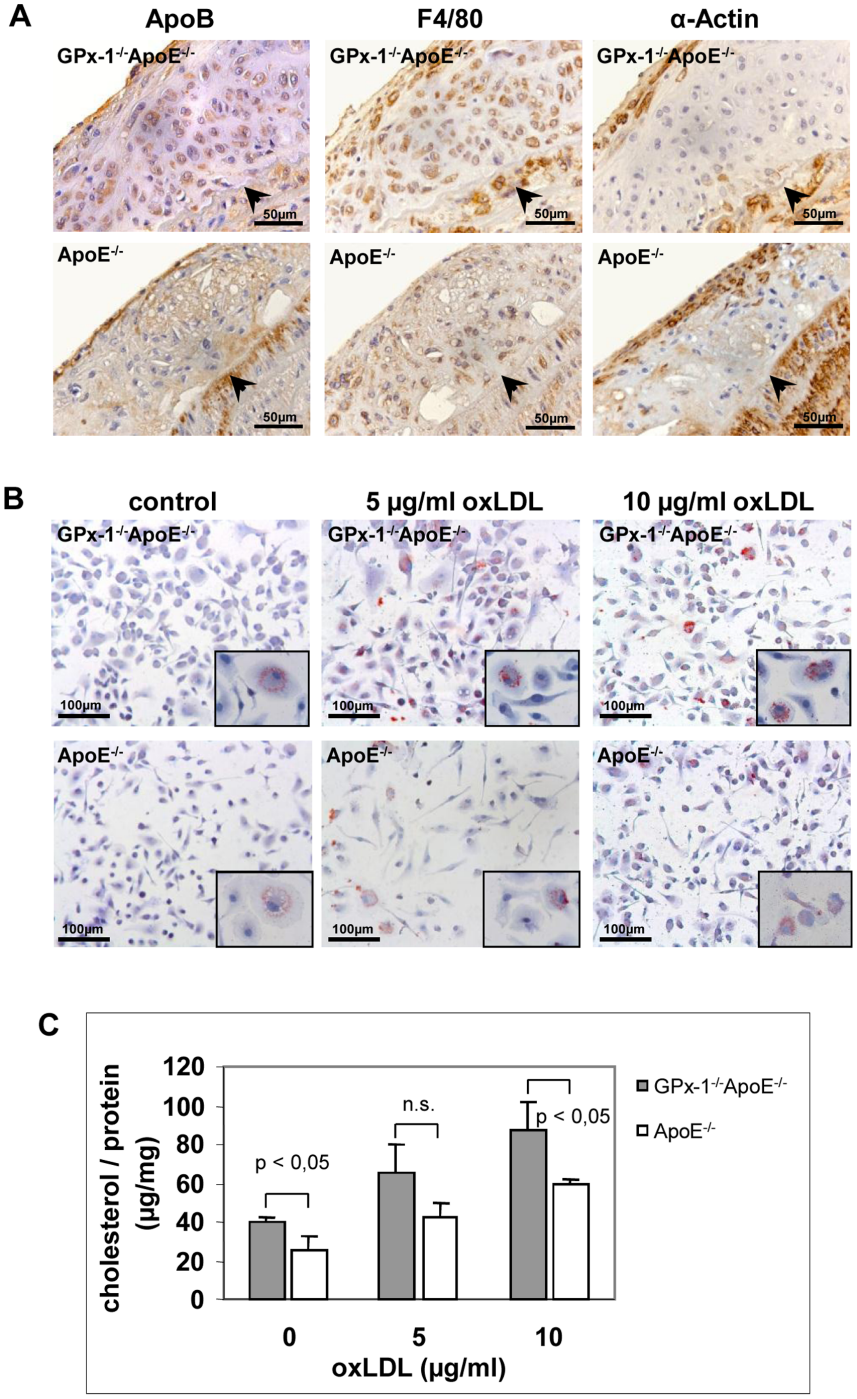
Lipoprotein staining and effect of GPx-1 deficiency on oxLDL induced foam cell formation. **A,** Immunohistochemical staining of lipoprotein apo B in parallel with staining of macrophages and SMCs in sequential sections of the aortic arch of both GPx-1^−/−^ApoE^−/−^ (upper panels) and ApoE^−/−^ (lower panels) mice. The vessel lumen is to the upper left-hand corner. The demarcation between intima and media is indicated by arrowheads. **B, C,** Effect of GPx-1 deficiency on oxLDL induced foam cell formation. After differentiation for 3 days with 10 ng/ml MCSF, mouse peritoneal macrophages were incubated with 5 and 10 µg/ml oxLDL, respectively, for 24 hours. **B,** Representative photomicrographs of peritoneal macrophages stained with oil-red O (magnification ×20, inserts ×100). **C,** Quantitative analysis of cellular cholesterol content in mouse peritoneal macrophages. After incubation with oxLDL, the cells were lysed and homogenized and total cholesterol content was quantified by fluorescence measurements. The results were expressed as total cholesterol per cellular protein. Each value represents the mean ± SD of four separate measurements.

To localize the cellular distribution of GPx-1 gene expression in mice atherosclerotic lesions, we performed *in situ*-hybridization and immunohistochemistry in lesions of the aortic sinus of GPx-1^−/−^ApoE^−/−^ and ApoE^−/−^ mice after 12 weeks on the WTD. GPx-1 mRNA expression was detected by *in situ*-hybridization and both macrophages and SMCs as the main cellular components of atherosclerotic lesions were detected by immunohistochemistry (see Materials and Methods). As shown in Figure 1 A and B, GPx-1 mRNA colocalizes with macrophage-rich areas and - even though to a much less extent - with SMCs. Furthermore, GPx-1 protein expression was detected by double staining for both GPx-1/F4/80 and GPx-1/α-Actin using a monoclonal GPx-1 antibody corroborating that both macrophages and SMCs contribute to GPx-1 expression within atherosclerotic lesions in ApoE^−/−^ mice (Fig. 1 C). Immunohistochemical staining of apo B revealed that both macrophages and SMCs - even though to a much less extent - contribute to foam cell formation in atherosclerotic lesions of GPx-1^−/−^ApoE^−/−^ and ApoE^−/−^ mice (Fig. 2 A).

### Effect of GPx-1 Deficiency on OxLDL Induced Macrophage Foam Cell Formation

To examine the effect of GPx-1 deficiency on oxLDL induced foam cell formation, isolated mouse peritoneal macrophages were differentiated for 3 days with 10 ng/ml MCSF and then incubated with 5 and 10 µg/ml oxLDL, respectively, for 24 hours. Staining with oil-red O showed accumulation of intracellular lipid droplets typical of foam cell formation. Peritoneal macrophages isolated from GPx-1^−/−^ApoE^−/−^ mice showed a qualitative tendency towards more extensive oil-red O staining compared with those isolated from ApoE^−/−^ mice (Figure 2 B). These findings were corroborated by quantitative analysis of cellular cholesterol content showing higher cellular cholesterol content of macrophages from GPx-1^−/−^ApoE^−/−^ mice that were incubated with 10 µg/ml oxLDL for 24 h compared with ApoE^−/−^ control mice (p<0,05; Figure 2 C). A similar, albeit statistically not significant trend could already be observed by incubation with 5 µg/ml oxLDL (Figure 2 C).

In an attempt to specify cellular uptake of oxLDL, quantitative real-time PCR for both scavenger receptors SRA and CD36 in GPx-1^−/−^ApoE^−/−^ compared with ApoE^−/−^ control mice was performed. By incubation with 20 µg/ml oxLDL and MCSF, relative expression of SRA1 mRNA (3±0,98 v.s. 1,33±0,53, p<0,05) but not CD36 mRNA (3,01±1,51 v.s. 3,02±1,68) was significantly increased in GPx-1^−/−^ApoE^−/−^ macrophages.

### Effect of GPx-1 Deficiency on OxLDL Induced Macrophage Proliferation

**Figure 3:**
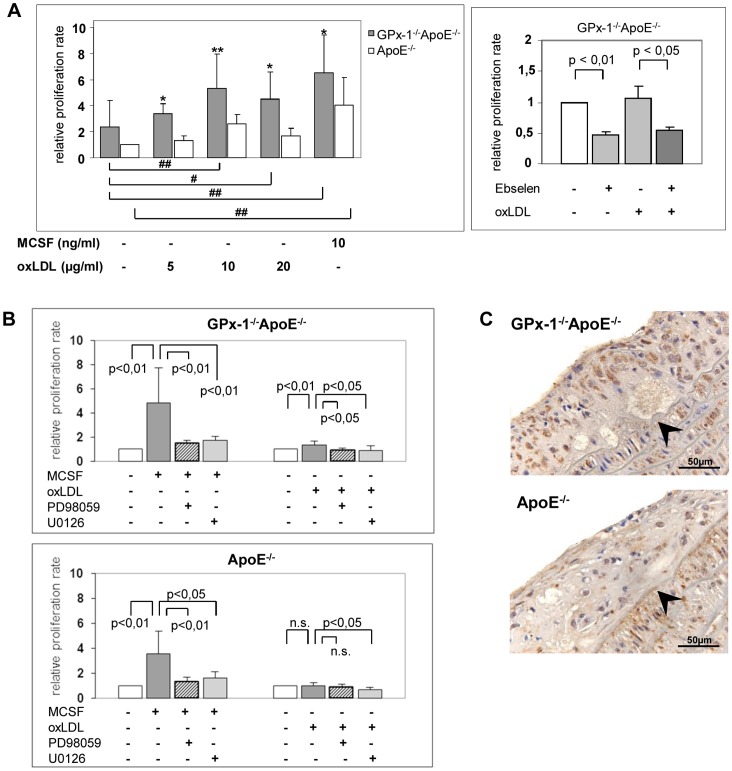
Effect of GPx-1 deficiency and MAPK signaling pathways inhibitors on macrophage proliferation. Macrophages were collected after differentiation of thioglycollate-elicited mouse peritoneal macrophages for 3 days in 10 ng/ml MCSF. **A left panel**, macrophages (2,5×10^4^ cells) were incubated with MCSF or oxLDL, respectively, and BrdU for another 48 hours. Subsequently, the proliferative activity was investigated with a BrdU-based chemiluminescence assay and expressed as relative proliferation rate relating to control cells from ApoE^−/−^ mice without stimulus. Data represent means ± SD of 4 to 5 separate experiments. *p<0.05 or **p<0,01 above the histogram indicate statistically significant differences between the different genotypes and below the histogram compared with cells without treatment of MCSF or oxLDL. **A right panel**, macrophages from GPx-1^−/−^ApoE^−/−^ mice were pretreated with 10 µM ebselen for 1 h and incubated with 10 µg/ml oxLDL and BrdU for another 48 h. The proliferation rate was expressed as the relative proliferation rate relating to the proliferative activity of untreated cells. Data represent means ± SD of 3 independent experiments. **B,** macrophages of GPx-1^−/−^ApoE^−/−^ (upper panel) and ApoE^−/−^ (lower panel) mice were pre-incubated with 75 µM PD98059 or 15 µM U0126, respectively, for 1 h and then incubated with 10 ng/ml MCSF (left) or 10 µg/ml oxLDL (right) and BrdU for another 48 h. The proliferation rate was expressed as the relative proliferation rate relating to the proliferative activity of untreated cells. Data represent means ± SD of 3 to 5 independent experiments. **C,** Representative immunohistochemical staining for the proliferation marker PCNA in atherosclerotic lesions of the aortic sinus demonstrating more pronounced positive nuclear staining in GPx-1^−/−^ApoE^−/−^ mice (left panel) than ApoE^−/−^ mice (right panel). The vessel lumen is to the upper left-hand corner. The demarcation between intima and media is indicated by arrowheads.

We investigated whether proliferative acitivity of monocyte-derived macrophages might account for the increased cellularity of early atherosclerotic lesions in GPx-1^−/−^ApoE^−/−^ mice [16]. The proliferation rate of mouse peritoneal macrophages was assessed with a BrdU-based chemiluminescence assay (Figure 3 A, left panel). MCSF (10 ng/ml) significantly induced the proliferation of macrophage from GPx-1^−/−^ApoE^−/−^ and ApoE^−/−^ control mice, respectively (p<0,01). Moreover, macrophages from GPx-1^−/−^ApoE^−/−^ mice showed significantly more BrdU incorporation than macrophages from ApoE^−/−^ control mice (p<0.05). Likewise, oxLDL had significant effects on macrophage proliferation at 10 and 20 µg/ml in GPx-1^−/−^ApoE^−/−^ mice and again, macrophages from GPx-1^−/−^ApoE^−/−^ mice showed significantly more BrdU incorporation than macrophages from ApoE^−/−^ control mice at any concentration of oxLDL (p<0,05). Interestingly, the GPx mimic ebselen led to a significant decrease of GPx-1^−/−^ApoE^−/−^ macrophage proliferation (p<0,05, Figure 3 A, right panel).

### Effects of MAPK Signaling Pathways Inhibitors on MCSF or OxLDL-Induced Macrophage Proliferation

The relationship between MCSF- or oxLDL-induced macrophage proliferation and the involvement of mitogen activated protein kinase (MAPK) pathways was further investigated by measuring BrdU incorporation into macrophages from GPx-1^−/−^ApoE^−/−^ and ApoE^−/−^ mice in the presence of MEK1/2 inhibitors PD98059 and U0126.

Pretreatment with both PD98059 and U0126 significantly inhibited both MCSF- (p<0,01) and oxLDL- (p<0,05) induced proliferation of peritoneal macrophages from GPx-1^−/−^ApoE^−/−^ mice (Figure 3 B, upper panel).

As for ApoE^−/−^ control mice (Figure 3 B, lower panel) statistical significance was reached for inhibition by both PD98059 and U0126 of MCSF-induced proliferation (p<0,01 and p<0,05, respectively) as well as U0126 for oxLDL-induced proliferation (p<0,05). Other differences were not statistically significant.

These results indicate that MCSF- or oxLDL-induced proliferation of peritoneal macrophages from GPx-1^−/−^ApoE^−/−^ mice is most susceptible to inhibition of the ERK1/2 signaling pathway. Finally, we corroborated these findings *in vivo* by immunohistochemistry for the proliferation marker PCNA demonstrating a significantly higher expression in atherosclerotic lesions of the aortic arch of GPx-1^−/−^ApoE^−/−^ mice (mean score: 1.88±0.49, n = 4) compared with ApoE^−/−^ control mice (mean score: 1.38±0.51, n = 3, p = 0.041; Fig. 3 C).

### Effects of MCSF and/or OxLDL on the Phosphorylation of MAPK

**Figure 4:**
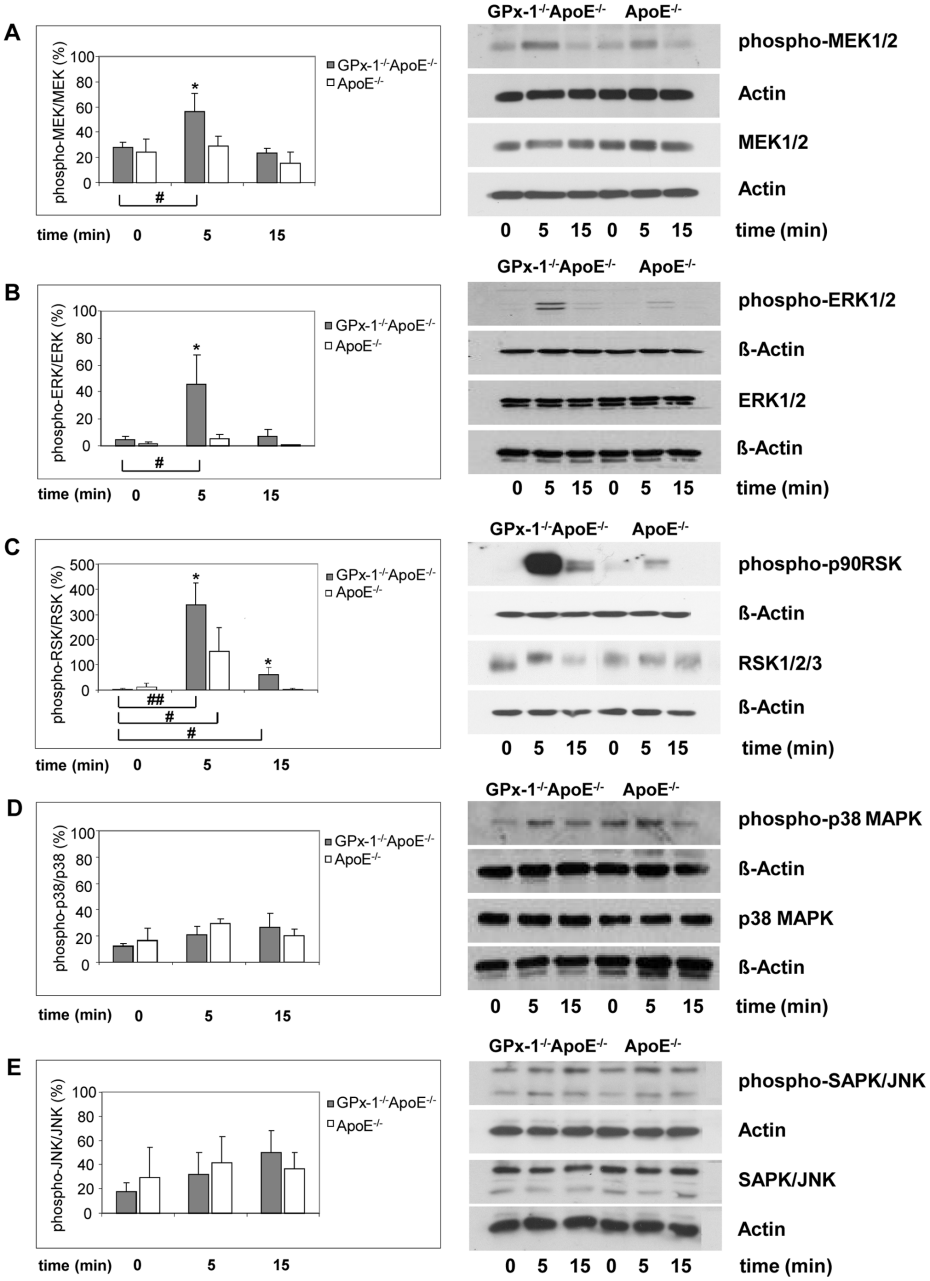
Effects of MCSF on the phosphorylation of MAPKs. After pre-incubation for 3 days with 10 ng/ml MCSF, peritoneal macrophages were incubated for 5 and 15 min with 10 ng/ml MCSF. Cellular protein was extracted and protein samples (0.4 mg/ml) were analyzed by Western blot with specific antibodies: anti-phosphorylated MEK1/2 or anti-MEK1/2 (**A,** right), anti-phosphorylated ERK1/2 or anti-ERK1/2 (**B,** right), anti-phosphorylated p90RSK or anti-RSK1/2/3 (**C**, right), anti-phosphorylated p38 MAPK or anti-p38 MAPK (**D,** right) and anti-phosphorylated SAPK/JNK or anti-SAPK/JNK (**E**, right) antibodies (representative experiments). ß-Actin or Actin were used as control. Quantitative results were calculated by band densitometry with the intensity of phosphorylated MEK1/2, ERK1/2, p90RSK, p38 MAPK and SAPK/JNK normalized to the total MEK1/2, ERK1/2, RSK1/2/3, p38 MAPK and SAPK/JNK (**A–E**, left panels). Data represent mean ± SD of 3 separate experiments. *, **indicate statistically significant differences (*p<0.05, **p<0.01) compared with cells without MCSF treatment.

To explain the effect of MCSF on MAPKs activation in macrophages from GPx-1 deficient mice, we determined the level of ERK1/2, p38 MAPK and SAPK/JNK phosphorylation in peritoneal macrophages using an antibody raised against both phosphorylation sites required for activation of ERK1/2 (Figure 4 B, right), p38 MAPK (Figure 4 D, right) and SAPK/JNK (Figure 4 E, right). MCSF induced early ERK1/2 phosphorylation significantly more in macrophages of GPx-1^−/−^ApoE^−/−^ mice than in ApoE^−/−^ control mice after 5 min (p<0,05). After 15 min this difference was no longer significant (Figure 4 B, left). MCSF did not increase p38 MAPK (Figure 4 D, left) and SAPK/JNK (Figure 4 E, left) phosphorylation significantly. Since ERK1/2 are activated by MEK1/2 and p90RSK is an important downstream substrate of ERK1/2, we tested further whether MEK1/2 and p90RSK are also activated by MCSF. Using an antibody reacting with phosphorylated MEK1/2 and p90RSK, MEK1/2 (Figure 4 A) and p90RSK (Figure 4 C), phosphorylation was found to be enhanced significantly in macrophages of GPx-1^−/−^ApoE^−/−^ mice compared with ApoE^−/−^ control mice at 5 min (p<0,05), and then MEK1/2 declined to basal level after 15 min.

**Figure 5:**
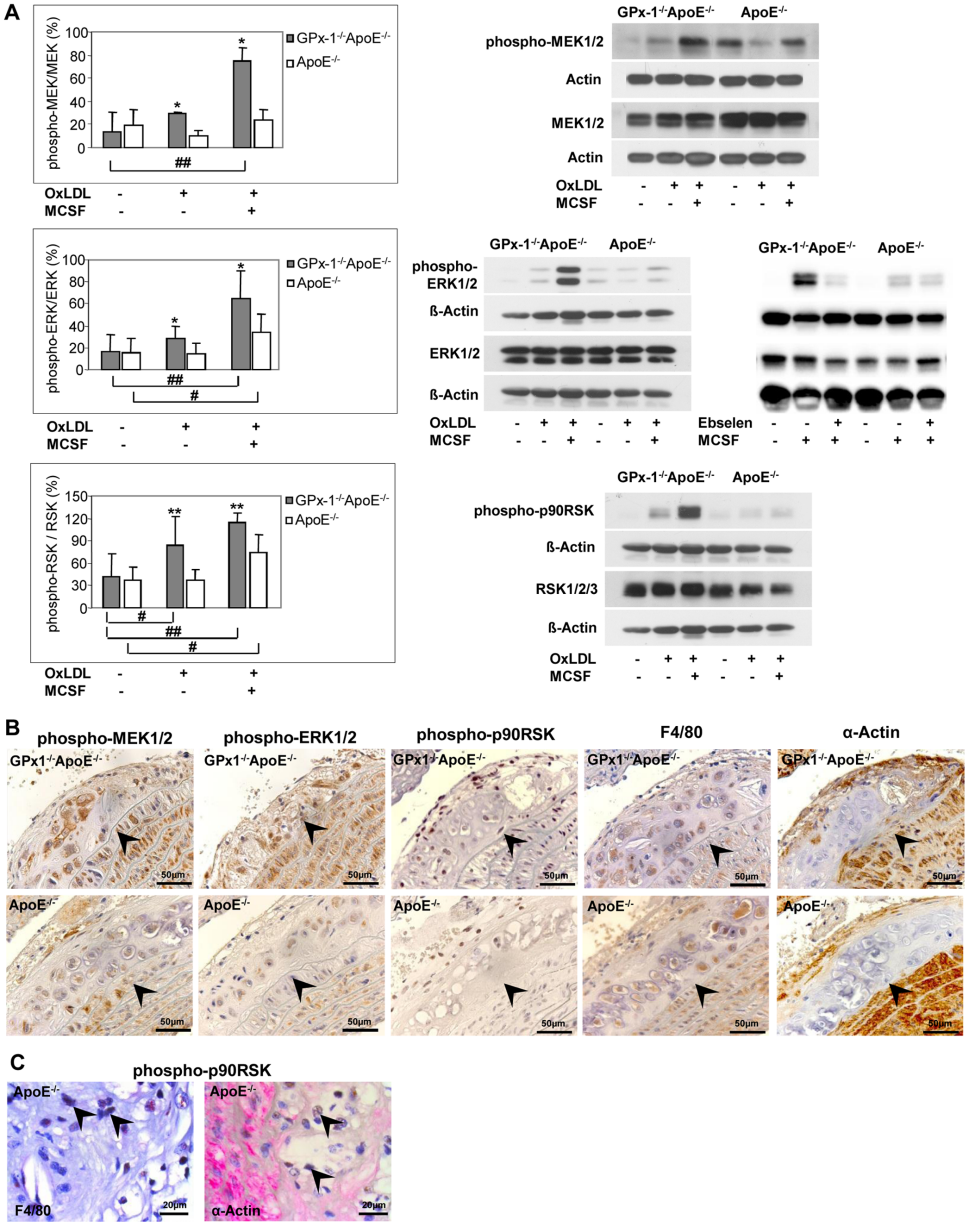
Effects of ebselen or oxLDL on MAPK phosphorylation and expression of MAPK in mice lesions. **A,** After pre-incubation for 3 days with 10 ng/ml MCSF, peritoneal macrophages were incubated for 5 min with 10 µg/ml oxLDL with or without MCSF or MCSF with or without ebselen. Cellular protein was extracted and protein samples (0.4 mg/ml) were analyzed by Western blot with specific antibodies: anti-phosphorylated MEK1/2 or anti-MEK1/2 (**A,** upper panel**,** right), anti-phosphorylated ERK1/2 or anti-ERK1/2 (**A,** middle panel**,** right) or anti-phosphorylated p90RSK or anti-RSK1/2/3 (**A,** lower panel, right) antibodies (representative experiments). ß-Actin or Actin were used as control. Quantitative results were calculated by band densitometry with the intensity of phosphorylated MEK1/2, ERK1/2, p90RSK normalized to total MEK1/2, ERK1/2, RSK1/2/3 (**A,** upper, middle and lower panel, left). Data represent mean ± SD of 5–7 separate experiments. * p<0.05 or ** p<0,01 above the histogram indicate statistically significant differences between the different genotypes and below the histogram compared with cells without treatment of MCSF or oxLDL. **B,** expression of phosphorylated MEK1/2 and ERK1/2 in parallel with staining of macrophages and SMCs in sequential sections of the aortic arch of both GPx-1^−/−^ApoE^−/−^ (upper panels) and ApoE^−/−^ (lower panels) mice. There is more pronounced expression of phosphorylated ERK1/2 and MEK1/2 both in macrophages and SMCs of GPx-1^−/−^ApoE^−/−^ compared with ApoE^−/−^ mice. **C**, representative double immunohistochemical staining for p-p90RSK (nuclei, brown), macrophages or SMCs (red) in ApoE^−/−^ mice demonstrating expression of p-p90RSK in macrophages rather than in SMCs (arrowheads). The vessel lumen is to the upper left-hand corner. The demarcation between intima and media is indicated by arrowheads.

These significant effects of GPx-1 deficiency were similar upon stimulation with oxLDL either with or without MCSF (Figure 5 A). In case of p90RSK, oxLDL alone even triggered increased phosphorylation in macrophages of GPx-1^−/−^ApoE^−/−^ compared with ApoE^−/−^ control mice which could not be observed in unstimulated cells (Figure 5 A, lower panel). Notably, the GPx mimic ebselen almost completely abrogated the phosphorylation of ERK in GPx-1^−/−^ApoE^−/−^ but had no effect on phosphorylation of ERK in ApoE^−/−^ control mice (Figure 5 A, middle panel).

To corroborate these *in vivo* findings, we representatively chose phosphorylated ERK1/2 for a more detailed analysis and performed representative immunohistochemistry both in ApoE^−/−^ and GPx-1^−/−^ApoE^−/−^ mice demonstrating a significantly higher expression in atherosclerotic lesions from the aortic arch of GPx-1^−/−^ApoE^−/−^ (mean score: 1.33±0.49, n = 5) compared with ApoE^−/−^ control mice (mean score: 0.83±0.39, n = 4, p = 0.034; Figure 5 B, upper and middle panels). Furthermore, serial and double immunohistochemical staining revealed that phopsphorylated MEK1/2 and ERK1/2 are expressed in both macrophages and SMCs, whereas phosphorylated p90RSK is expressed in macrophages rather than in SMCs (Figure 5 C).

## Discussion

The present study extends our previous results on atherosclerosis development in GPx-1^−/−^ApoE^−/−^ mice [16] on the cellular level. Addressing the aims (1) to identify the cellular distribution of GPx-1 within atherosclerotic lesions and (2) to determine whether a lack of GPx-1 affects foam cell formation and known signal transduction pathways implicated in cellular proliferation, the following results have been provided: Within atherosclerotic lesions, both macrophages and - even to a much less extent - SMCs contribute to GPx-1 expression. GPx-1 deficiency increased oxLDL induced foam cell formation and led to increased proliferative activity of peritoneal macrophages. The MCSF- and oxLDL-induced proliferation of peritoneal macrophages from GPx-1^−/−^ApoE^−/−^ mice is mediated by the p44/42 MAPK signaling pathway as demonstrated by both ERK1/2 signaling pathways inhibitors and phosphorylation of ERK1/2, MEK1/2 and p90RSK.

To reasonably estimate the role of antioxidant enzymes during atherosclerotic lesion development, data concerning expression levels and localization of these enzymes are indispensable. By extraction of RNA from the aortic arch and descending aorta, and measurement of mRNA expression of pro- and antioxidant enzymes with real-time PCR, ‘t Hoen et al. [13] demonstrated that the aorta of apoE-deficient mice responds to atherogenic stimuli by a prelesional increase and subsequent decrease of the expression of antioxidant enzymes. Due to the methodology used, the cellular source of GPx-1 mRNA could not be identified. It was realized, however, that the expression of several antioxidant enzymes appeared to be positively correlated to CD68 expression levels, whereas at 12 weeks, they tend to be negatively correlated [13]. As we have now demonstrated that GPx-1 expression is found in lesional macrophages and as the relative number of macrophages in atherosclerotic lesions decreases during atherosclerotic lesion progression [16], it is evident that macrophage infiltration is at least partly responsible for the initial induction and subsequent decrease of GPx-1 expression levels reported by ‘t Hoen et al [13]. Consistently with a previous report [23] and illustrated by double immunohistochemical staining for the different antigens (Fig. 1 C), SMCs also contribute to GPx-1 expression within our mice atherosclerotic lesions, even to a much less extent. The validity of our animal model was demonstrated convincingly by the lack of hybridization of the antisense GPx-1 cRNA to the tissue sections of GPx-1^−/−^ApoE^−/−^ mice. Taken together, our results point to the need of precise localization of mRNA expression in tissue sections by *in situ*-hybridization to avoid misinterpretation of the results obtained by mere quantitative real-time PCR of entire tissue homogenates.

After having identified macrophages as an important cellular source of GPx-1 expression within atherosclerotic lesions, we subsequently focused on this cell type to determine whether a lack of GPx-1 impacts on foam cell formation and known signal transduction pathways implicated in cellular proliferation. However, the contribution of SMCs to foam cell formation should be recognized and is illustrated by colocalization of apo B and α-actin staining in atherosclerotic lesions of both GPx-1^−/−^ApoE^−/−^ and ApoE^−/−^ mice (Fig. 2 A). Likewise, in a nice study on lesion development in apoE-deficient mice, Nakashima et al. demonstrated that, as the lesions continued to progress with increasing age (20 weeks of age like in our study), SMCs appeared, many of which contained lipid deposits [24].

First of all, we were able to demonstrate a significant cumulative effect of GPx-1 deficiency on oxLDL induced foam cell formation. These data add to the reports of other investigators reporting that both LDL oxidation and oxLDL-induced apoptosis were significantly increased when LDL was incubated with aortic segments and SMCs obtained from GPx-1^−/−^ mice [10]. Unlike Guo et al. [10], however, the macrophages in our study were incubated with *in vitro* oxidized LDL, showing that there is also a remarkable impact of GPx-1 deficiency on cellular uptake of this modified lipoprotein. Additionally, the heterogeneity in terms of lipid uptake comparing one cell with another might be due to the quite low oxLDL concentrations we used and/or the well known different phenotypes of mouse monocytes [25], [26]. As suggested by quantitative real-time PCR, the scavenger receptor SRA1 but not CD36 is upregulated in GPx-1^−/−^ ApoE^−/−^ mice suggesting that the former is intimately involved in the increased cellular uptake of oxLDL.

GPx-1 deficiency was always associated with an increased proliferative rate of macrophages independent whether cells were incubated with MCSF ([16] and Figure 3 A) or with oxLDL even at low concentrations (5 and 10 µg/ml). This points to the important impact of oxidative stress on macrophage proliferation caused by the lack of GPx-1. In contrast, in several earlier studies on macrophages without GPx-1 deficiency, a significant effect of oxLDL on macrophage proliferation was observed not until 20 µg/ml [27], [28], [29], [30], [31]. Another representative example of the cumulative effect of oxLDL on oxidative stress induced by GPx-1 deficiency is the phosphorylation of p90RSK, an important downstream substrate of ERK1/2 (Figure 5 A). That GPx-1 deficiency indeed leads to increased reactive oxygen species in the aortic wall as well as increased overall oxidative stress has been already elucidated by functional experiments in our previous study [7]. The fact that the impact of GPx-1 deficiency on macrophage proliferation could be abolished by the GPx mimic ebselen (Figs. 3 A, 5 A) opens novel avenues of therapeutic strategies against atherosclerosis. In future studies it may be interesting to further investigating this issue by determining whether the effect of GPx-1 deficiency can be abolished by applying siRNA technology to knock down the key proteins or pathways or by investigating transgenic mice overexpressing GPx-1 [32].

How are the extracellular MCSF or oxLDL signals transduced to cellular proliferative responses? Among the prime suspects are members of the MAPK family. MAPK cascades have been shown to play a key role in transduction of extracellular signals to cellular responses. Three major subfamilies of structurally related MAPK have been identified in mammalian cells, which are termed p44/42 MAPK (extracellular-signal regulated kinase 1/2; ERK1/2), p38 MAPK (p38 mitogen activated protein kinase) and JNK/SAPKs (Jun N-terminal kinases/stress-activated protein kinase) [33]. Exploring the role of MAPK activation in MCSF- and oxLDL-induced macrophage proliferation and the effect of GPx-1 on the MAPK signaling pathway we found that macrophage proliferation of GPx-1^−/−^ApoE^−/−^ mice is susceptible to inhibition of the ERK1/2 signaling pathway. This observation is in line with previous reports of oxidative stress-induced ERK1/2 activation in a variety of cell types [34], [35], [36], [37], [38].

To explain the effect of MCSF and/or oxLDL on MAPK activation in macrophages from GPx-1 deficient mice, we determined the levels of MEK1/2, ERK1/2, p90RSK, p38 MAPK and SAPK/JNK phosphorylation in MCSF- and/or oxLDL-induced peritoneal macrophages by Western blot analysis. In accordance with the inhibition experiments, a significant short-term phosphorylation of both MEK1/2 and ERK1/2 was detected as early as five minutes after stimulation with MCSF in GPx-1 deficient macrophages. These significant effects of GPx-1 deficiency were similar upon stimulation with oxLDL either with or without MCSF. In particular, there was a striking phosphorylation of 90 kDa ribosomal S6 kinases (p90RSK) after stimulation with MCSF and even triggered by oxLDL alone. p90RSK was among the first substrates of ERK1/2 to be discovered and has proven to be an ubiquitous and versatile mediator of ERK1/2 signal transduction inducing diverse biological functions including regulation of nucleosome and gene expression, mRNA stability and translation, and cell proliferation and survival [39].

Since ERK1/2 are activated by MEK1/2 and p90RSK is an important downstream substrate of ERK1/2, our results corroborate the susceptibility of the p44/42 MAPK (ERK1/2) signaling pathway to oxidative stress caused by GPx-1 deficiency. The *in vivo* significance of these results was illustrated by immunohistochemical findings demonstrating pronounced expression of phosphorylated ERK1/2, MEK1/2 and p90RSK in atherosclerotic lesions of GPx-1^−/−^ApoE^−/−^ mice. ERK1/2 expression and activation could also be observed in atherosclerotic lesions of cholesterol-fed rabbits, and phosphorylated ERK1/2 colocalizes with the proliferating cells including macrophages [40].

While we could not demonstrate an effect of GPx-1 deficiency on p38 MAPK, GPx-1 expression and/or induction has been shown to induce p38 MAPK in human endothelial cells [41], to abolish hypoxic activation of p38 MAPK induction in mouse embryonic fibroblasts [42], or to have no effect on p38 MAPK in human MCF-7 cells [43]. As for JNK, low levels of GPx-1 activity in selenium deficient mouse liver have been shown to induce JNK activation [44] while studies in human aortic endothelial cells have demonstrated abrogation of H_2_O_2_-induced increases in JNK by the GPx-1 mimic ebselen [45]. Taken together, these divergent results suggest that the susceptibility of the p38 MAPK and JNK/SAPKs pathways to GPx-1 obviously largely depends on the cell type investigated.

In conclusion, our present study demonstrates that GPx-1 deficiency has a significant impact on macrophage foam cell formation and proliferation via the p44/42 MAPK (ERK1/2) pathway encouraging further studies on new therapeutic strategies against atherosclerosis.

## References

[1] Arthur JR (2000) The glutathione peroxidases. Cell Mol Life Sci 57: 1825–1835. 11215509 10.1007/PL00000664 PMC11147127

[2] Flohe L (1988) Glutathione peroxidase. Basic Life Sci 49: 663–668. 3074794 10.1007/978-1-4684-5568-7_104

[3] Sies H (1999) Glutathione and its role in cellular functions. Free Radic Biol Med 27: 916–921. 10569624 10.1016/s0891-5849(99)00177-x

[4] Lubos E, Loscalzo J, Handy DE (2011) Glutathione peroxidase-1 in health and disease: from molecular mechanisms to therapeutic opportunities. Antioxid Redox Signal 15: 1957–1997. 21087145 10.1089/ars.2010.3586 PMC3159114

[5] Fu Y, Porres JM, Lei XG (2001) Comparative impacts of glutathione peroxidase-1 gene knockout on oxidative stress induced by reactive oxygen and nitrogen species in mouse hepatocytes. Biochem J 359: 687–695. 11672444 10.1042/0264-6021:3590687 PMC1222191

[6] Fu Y, Sies H, Lei XG (2001) Opposite roles of selenium-dependent glutathione peroxidase-1 in superoxide generator diquat- and peroxynitrite-induced apoptosis and signaling. J Biol Chem 276: 43004–43009. 11562367 10.1074/jbc.M106946200

[7] Torzewski M, Ochsenhirt V, Kleschyov AL, Oelze M, Daiber A, et al (2007) Deficiency of glutathione peroxidase-1 accelerates the progression of atherosclerosis in apolipoprotein E-deficient mice. Arterioscler Thromb Vasc Biol 27: 850–857. 17255533 10.1161/01.ATV.0000258809.47285.07

[8] De Haan JB, Crack PJ, Flentjar N, Annello RC, Hertzog PJ, et al (2003) An imbalance in antioxidant defense affects cellular function: the pathophysiological consequences of a reduction in antioxidant defense in the glutathione peroxidase-1 (Gpx1) knockout mouse. Redox Rep 8: 69–79. 12804009 10.1179/135100003125001378

[9] Steinberg D (1997) Low density lipoprotein oxidation and its pathobiological significance. J Biol Chem 272: 20963–20966. 9261091 10.1074/jbc.272.34.20963

[10] Guo Z, Van Remmen H, Yang H, Chen X, Mele J, et al (2001) Changes in expression of antioxidant enzymes affect cell-mediated LDL oxidation and oxidized LDL-induced apoptosis in mouse aortic cells. Arterioscler Thromb Vasc Biol 21: 1131–1138. 11451741 10.1161/hq0701.092092

[11] Blankenberg S, Rupprecht HJ, Bickel C, Torzewski M, Hafner G, et al (2003) Glutathione peroxidase 1 activity and cardiovascular events in patients with coronary artery disease. N Engl J Med 349: 1605–1613. 14573732 10.1056/NEJMoa030535

[12] Lapenna D, de Gioia S, Ciofani G, Mezzetti A, Ucchino S, et al (1998) Glutathione-related antioxidant defenses in human atherosclerotic plaques. Circulation 97: 1930–1934. 9609086 10.1161/01.cir.97.19.1930

[13] ‘t Hoen PA, Van der Lans CA, Van Eck M, Bijsterbosch MK, Van Berkel TJ, et al (2003) Aorta of ApoE-deficient mice responds to atherogenic stimuli by a prelesional increase and subsequent decrease in the expression of antioxidant enzymes. Circ Res 93: 262–269. 12829615 10.1161/01.RES.0000082978.92494.B1

[14] Forgione MA, Cap A, Liao R, Moldovan N, Eberhardt RT, et al (2002) Heterozygous cellular glutathione peroxidase deficiency in the mouse: abnormalities in vascular and cardiac function and structure. Circulation 2002 Aug 27 106: 1154–1158. 10.1161/01.cir.0000026820.87824.6a 12196344

[15] Dayal S, Brown KL, Weydert CJ, Oberley LW, Arning E, et al (2002) Deficiency of glutathione peroxidase-1 sensitizes hyperhomocysteinemic mice to endothelial dysfunction. Arterioscler Thromb Vasc Biol 22: 1996–2002. 12482825 10.1161/01.atv.0000041629.92741.dc

[16] Torzewski M, Ochsenhirt V, Kleschyov AL, Oelze M, Daiber A, et al (2007) Deficiency of glutathione peroxidase-1 accelerates the progression of atherosclerosis in apolipoprotein E-deficient mice. Arterioscler Thromb Vasc Biol 27: 850–857. 17255533 10.1161/01.ATV.0000258809.47285.07

[17] Lewis P, Stefanovic N, Pete J, Calkin AC, Giunti S, et al (2007) Lack of the antioxidant enzyme glutathione peroxidase-1 accelerates atherosclerosis in diabetic apolipoprotein E-deficient mice. Circulation 115(16): 2178–2187. 17420349 10.1161/CIRCULATIONAHA.106.664250

[18] Paigen B, Morrow A, Holmes PA, Mitchell D, Williams RA (1987) Quantitative assessment of atherosclerotic lesions in mice. Atherosclerosis 68(3): 231–240. 3426656 10.1016/0021-9150(87)90202-4

[19] Mach F, Schonbeck U, Sukhova GK, Atkinson E, Libby P (1998) Reduction of atherosclerosis in mice by inhibition of CD40 signalling. Nature 394: 200–203. 9671306 10.1038/28204

[20] Bhakdi S, Torzewski M, Paprotka K, Schmitt S, Barsoom H, et al (2004) Possible protective role for C-reactive protein in atherogenesis: complement activation by modified lipoproteins halts before detrimental terminal sequence. Circulation 109: 1870–1876. 15037531 10.1161/01.CIR.0000124228.08972.26

[21] Wieland E, Dorweiler B, Bonitz U, Lieser S, Walev I, et al (1999) Complement activation by oxidatively modified low-density lipoproteins. Eur J Clin Invest 29: 835–841. 10583425 10.1046/j.1365-2362.1999.00548.x

[22] Wang H, Wang H, Xiong W, Chen Y, Ma Q, et al (2006) Evaluation on the phagocytosis of apoptotic spermatogenic cells by Sertoli cells in vitro through detecting lipid droplet formation by Oil Red O staining. Reproduction 132: 485–492. 16940289 10.1530/rep.1.01213

[23] Takapoo M, Chamseddine AH, Bhalla RC, Miller FJ Jr (2011) Glutathione peroxidase-deficient smooth muscle cells cause paracrine activation of normal smooth muscle cells via cyclophilin A. Vascul Pharmacol. 55: 143–148. 10.1016/j.vph.2011.07.002 PMC3218252 21782974

[24] Nakashima Y, Plump AS, Raines EW, Breslow JL, Ross R (1994) ApoE-deficient mice develop lesions of all phases of atherosclerosis throughout the arterial tree. Arterioscler Thromb 14: 133–140. 8274468 10.1161/01.atv.14.1.133

[25] Swirski FK, Libby P, Aikawa E, Alcaide P, Luscinskas FW, et al (2007) Ly-6Chi monocytes dominate hypercholesterolemia-associated monocytosis and give rise to macrophages in atheromata. J Clin Invest 117: 195–205. 17200719 10.1172/JCI29950 PMC1716211

[26] Tacke F, Alvarez D, Kaplan TJ, Jakubzick C, Spanbroek R, et al (2007) Monocyte subsets differentially employ CCR2, CCR5, and CX3CR1 to accumulate within atherosclerotic plaques. J Clin Invest 117: 185–194. 17200718 10.1172/JCI28549 PMC1716202

[27] Senokuchi T, Matsumura T, Sakai M, Matsuo T, Yano M, et al (2004) Extracellular signal-regulated kinase and p38 mitogen-activated protein kinase mediate macrophage proliferation induced by oxidized low-density lipoprotein. Atherosclerosis 176: 233–245. 15380445 10.1016/j.atherosclerosis.2004.05.019

[28] Hamilton JA, Myers D, Jessup W, Cochrane F, Byrne R, et al (1999) Oxidized LDL can induce macrophage survival, DNA synthesis, and enhanced proliferative response to CSF-1 and GM-CSF. Arterioscler Thromb Vasc Biol 19: 98–105. 9888871 10.1161/01.atv.19.1.98

[29] Sakai M, Miyazaki A, Hakamata H, Sasaki T, Yui S, et al (1994) Lysophosphatidylcholine plays an essential role in the mitogenic effect of oxidized low density lipoprotein on murine macrophages. J Biol Chem 269 (50): 31430–31435. 7989310

[30] Matsumura T, Sakai M, Kobori S, Biwa T, Takemura T, et al (1997) Two intracellular signaling pathways for activation of protein kinase C are involved in oxidized low-density lipoprotein-induced macrophage growth. Arterioscler Thromb Vasc Biol 17(11): 3013–3020. 9409288 10.1161/01.atv.17.11.3013

[31] Martens JS, Reiner NE, Herrera-Velit P, Steinbrecher UP (1998) Phosphatidylinositol 3-kinase is involved in the induction of macrophage growth by oxidized low density lipoprotein. J Biol Chem 273(9): 4915–4920. 9478935 10.1074/jbc.273.9.4915

[32] Mirochnitchenko O, Palnitkar U, Philbert M, Inouye M (1995) Thermosensitive phenotype of transgenic mice overproducing human glutathione peroxidases. Proc Natl Acad Sci U S A 92: 8120–8124. 7667255 10.1073/pnas.92.18.8120 PMC41107

[33] Zhang W, Liu HT (2002) MAPK signal pathways in the regulation of cell proliferation in mammalian cells. Cell Res 12: 9–18. 11942415 10.1038/sj.cr.7290105

[34] Jiménez LA, Zanella C, Fung H, Janssen YM, Vacek P, et al (1997) Role of extracellular signal-regulated protein kinases in apoptosis by asbestos and H2O2. Am J Physiol 273: 1029–1035. 10.1152/ajplung.1997.273.5.L1029 9374731

[35] Buder-Hoffmann S, Palmer C, Vacek P, Taatjes D, Mossman B (2001) Different accumulation of activated extracellular signal-regulated kinases (ERK 1/2) and role in cell-cycle alterations by epidermal growth factor, hydrogen peroxide, or asbestos in pulmonary epithelial cells. Am J Respir Cell Mol Biol 24: 405–413. 11306433 10.1165/ajrcmb.24.4.4290

[36] Kim BY, Han MJ, Chung AS (2001) Effects of reactive oxygen species on proliferation of Chinese hamster lung fibroblast (V79) cells. Free Radic Biol Med 30: 686–698. 11295367 10.1016/s0891-5849(00)00514-1

[37] Blanc A, Pandey NR, Srivastava AK (2003) Synchronous activation of ERK 1/2, p38mapk and PKB/Akt signaling by H2O2 in vascular smooth muscle cells: potential involvement in vascular disease (review). Int J Mol Med 11: 229–234. 12525883

[38] Conde de la Rosa L, Schoemaker MH, Vrenken TE, Buist-Homan M, Havinga R, et al (2006) Superoxide anions and hydrogen peroxide induce hepatocyte death by different mechanisms: involvement of JNK and ERK MAP kinases. J Hepatol 44: 918–929. 16310883 10.1016/j.jhep.2005.07.034

[39] Cargnello M, Roux PP (2011) Activation and function of the MAPKs and their substrates, the MAPK-activated protein kinases. Microbiol Mol Biol Rev 75 (1): 50–83. 10.1128/MMBR.00031-10 PMC3063353 21372320

[40] Hu Y, Dietrich H, Metzler B, Wick G, Xu Q (2000) Hyperexpression and activation of extracellular signal-regulated kinases (ERK1/2) in atherosclerotic lesions of cholesterol-fed rabbits. Arterioscler Thromb Vasc Biol 20 (1): 18–26. 10.1161/01.atv.20.1.18 10634796

[41] Wagner AH, Kautz O, Fricke K, Zerr-Fouineau M, Demicheva E, et al (2009) Upregulation of glutathione peroxidase offsets stretch-induced proatherogenic gene expression in human endothelial cells. Arterioscler Thromb Vasc Biol 29: 1894–1901. 19729606 10.1161/ATVBAHA.109.194738

[42] Emerling BM, Platanias LC, Black E, Nebreda AR, Davis RJ, et al (2005) Mitochondrial reactive oxygen species activation of p38 mitogen-activated protein kinase is required for hypoxia signaling. Mol Cell Biol 25: 4853–4862. 15923604 10.1128/MCB.25.12.4853-4862.2005 PMC1140591

[43] Nasr MA, Fedele MJ, Esser K, Diamond AM (2004) GPx-1 modulates Akt and P70S6K phosphorylation and Gadd45 levels in MCF-7 cells. Free Radic Biol Med 37: 187–195. 15203190 10.1016/j.freeradbiomed.2004.04.038

[44] Cheng WH, Zheng X, Quimby FR, Roneker CA, Lei XG (2003) Low levels of glutathione peroxidase 1 activity in selenium-deficient mouse liver affect c-Jun N-terminal kinase activation and p53 phosphorylation on Ser-15 in pro-oxidant-induced aponecrosis. Biochem J 370: 927–934. 12492400 10.1042/BJ20021870 PMC1223242

[45] Chew P, Yuen DY, Koh P, Stefanovic N, Febbraio MA, et al (2009) Site-specific antiatherogenic effect of the antioxidant ebselen in the diabetic apolipoprotein E-deficient mouse. Arterioscler Thromb Vasc Biol 29: 823–830. 19325139 10.1161/ATVBAHA.109.186619

